# The social-devaluation effect: interactive evaluation deteriorates likeability of objects based on daily relationship

**DOI:** 10.3389/fpsyg.2014.01558

**Published:** 2015-01-09

**Authors:** Atsunori Ariga

**Affiliations:** Faculty of Psychology, Rissho UniversityTokyo, Japan

**Keywords:** social-devaluation effect, social interaction, co-evaluation, likeability, preference

## Abstract

Although previous research has explored the effects of discussion on optimal and collective group outcomes, it is unclear how an individual’s preference for an object is modulated by discussion with others. This study investigated the determinants of likeability ratings under two conditions. In Experiment 1, pairs of participants consisting of friends evaluated various photographic images. Under the interactive condition, the participants discussed their impressions of each image for 30 s and then independently rated how much they liked it. Under the non-interactive condition, the participants did not interact with each other but instead only thought about their impressions of each image for 30 s before rating its likeability. The results indicate that the exchange of impressions between the participants affected the individual likeability ratings of objects. More specifically, the interactive participants generally rated the images as less likeable than did the non-interactive participants (*social-devaluation effect*). However, in Experiment 2, the effect was eliminated when the pairs consisted of strangers. These findings suggest that shared information modulates individual preferences but only when a daily relationship exists within a group.

## INTRODUCTION

People frequently evaluate objects in daily life. For example, humans evaluate artwork, movies, landscapes, friends, partners, and so on. In daily shopping, the evaluation of the likeability of goods or services critically determines consumers’ decision making during a purchase. Thus far, many cognitive psychologists have reported that likeability ratings given to objects are determined by the characteristics of the objects themselves or by one’s personal experience with them. For example, the likeability of symmetrical objects (e.g., Rhodes, 2006), canonically viewed objects (e.g., Palmer et al., 1981), objects that are targets of viewing (Shimojo et al., 2003), and familiar or novel objects (e.g., Zajonc, 1968; Park et al., 2010) are generally given high ratings.

Although the factors that consistently influence the likeability of an object have been explored under controlled conditions, likeability probably varies according to the social situation. Classical balance theory (Heider, 1958), which underscores the importance of maintaining cognitive consistency, asserts that one’s attitudes toward an object are modulated by one’s attitudes toward others as well as by the attitudes of others toward the same object. Furthermore, although attitudes toward an object have been traditionally regarded as stable evaluative representations that are stored in long-term memory, many studies have suggested that attitudes are not merely retrieved from memory but are constructed on the spot (e.g., Schwarz and Bohner, 2001; see also Gawronski and Bodenhausen, 2006). Apparently, a dynamic mechanism underlies the process by which humans evaluate objects in realistic social contexts. In fact, in daily life, objects are more frequently evaluated in the presence than in the absence of others (e.g., exchanging impressions of a movie with friends). Therefore, this study investigated how co-evaluation with others affects the likeability ratings given to objects.

Many studies evaluating group decision making in the fields of social and organizational psychology have traditionally explored how optimal and collective group outcomes are produced via discussion, a topic that is also of interest in economics and political science (e.g., Black, 1958; Smoke and Zajonc, 1962; Arrow, 1963; Davis, 1973; Fishburn, 1973; Ordeshook, 1973). Taking the most famous example, Stasser and Titus (1985) examined the effects of shared and unshared information on small-group decision making. In their experiment, each small group consisting of four members was required to choose one of three political candidates based on profile information about the candidates. Under the shared condition, all information about the three candidates was shared by all group members. Under the unshared condition, some information was shared by all members, but other information was divided among the individual members and not shared. These researchers found that under the shared condition, the participants preferred candidate A (i.e., a superior alternative). In contrast, under the unshared condition, where only negative information about candidate A was shared among the group, participants tended to prefer another candidate prior to and after the group discussion. However, this phenomenon, which is known as the common knowledge effect (Gigone and Hastie, 1996), is attenuated when discussion time is extended because the unshared information becomes more prevalent or dispersed (i.e., shared) over time (Larson et al., 1994; Schulz-Hardt et al., 2006; Scholten et al., 2007). It has also been reported that a group decision in favor of a superior alternative is impaired when information exchanged during the discussion is not attended to (Mojzisch and Schulz-Hardt, 2010). Although the manner in which information is sampled and pooled during group discussion remains controversial, there is a general consensus that discussion (or co-evaluation) plays a critical role in group decision making.

That said, when it comes to real-world consumer behavior, an individual typically expresses a preference about which item to purchase independently following discussion with friends or family instead of arriving at a collective decision based on the will of the group. Furthermore, superior alternatives do not always exist, and one’s attitudes toward given alternatives may be ambiguous, especially when one is eager to co-evaluate the alternatives with others (or ask others’ advice on the alternatives). Thus, it is necessary to examine how an individual’s preference for an object is modulated by discussion with others in which a variety of sometimes ambiguous impressions may be conveyed among individuals. The purpose of this study was to reveal the nature of the co-evaluation of likeability by demonstrating its effect on individual judgments about target images. Clarification of the manner in which objects are co-evaluated in realistic social contexts will provide a better understanding of human cognition.

In this study, pairs of participants interactively discussed a sequence of photographic images of objects and independently rated the likeability of each. One prediction is that analogous to group decision making, interactive evaluation influences likeability ratings in any direction, even when they are reported individually. The other prediction is that interactive evaluation does not influence the individual reports of likeability ratings, which means that discussion could be influential only when a group makes collective outcomes.

According to the previous studies (Brodbeck et al., 2002; Schulz-Hardt et al., 2006), the effect of shared information is stronger in homogeneous groups whose members share the same initial preferences prior to group discussion than in heterogeneous groups. If so, the influence of interactive evaluation, if any, could be modulated by the type of target images in this study. In other words, likeability ratings may be influenced by interactive evaluation more strongly for those target images that elicit relatively consistent initial preferences across participants than for those that elicit inconsistent (or ambiguous) initial preferences across participants.

## EXPERIMENT 1

### METHOD

#### Ethics statement

All experiments were approved by the internal review board of Department of Psychology, Rissho University. Written informed consent was obtained from each participant before the experiment.

#### Participants

The sample consisted of 30 pairs of naïve volunteers (60 undergraduates in total, 15 males, 20–24 years old); all pairs consisted of friends.

#### Stimuli

The stimuli were 13 photographic images of objects belonging to various categories: two male faces, two female faces, two animals, two buildings, two nature scenes, one depiction of food, one image of furniture, and one abstract painting. These images were selected from the database of international affective picture system (IAPS; Lang et al., 2008) for the sake of investigating whether the effect of co-evaluation, if any, depends on image category. Furthermore, in order to investigate whether the effect is modulated by the degree of likeability of the images, I selected a slightly positive image (0.90–1.10 higher than neutral point 5.00) and a slightly negative image (0.9–1.10 lower than neutral point) for each male, female, animal, building, and nature scene. Because most images of food, furniture, and abstract paintings were rated around neutral point, I selected a neutral image for each of them (4.90–5.10). Each image was printed in color on A4 paper.

#### Procedure

Participants sat across from one another at a table. In front of each participant, the full-color printed image was presented on the table by the experimenter. Participants’ task was to rate the likeability concerning the contents depicted in the image, not the abstract concept of them, using a 7-point Likert scale (1 = dislike, 7 = like). Under the interactive condition (*N* = 15 pairs), the two participants discussed their impression of each image for 30 s, which were timed with a stop-watch. Then, the experimenter flipped the image over and the participants rated how much they liked it using individual response sheets. Importantly, the pairs were told in advance that they did not have to agree about their ratings, and they were prohibited from talking about their actual ratings. After their ratings, the next images were presented. Under the non-interactive control condition (*N* = 15 pairs), the two participants did not interact with each other but independently thought about their impression of each image for 30 s before rating the likeability of the image.

Under both conditions, the participants’ faces were visible to their partners during the experiment, but both partners’ actual responses were physically hidden by boxes. The conversation under the interactive condition was recorded during the experiment. The participants performed 13 trials in total. The order of images was randomized across pairs.

### RESULTS

#### General results

**FIGURE 1:**
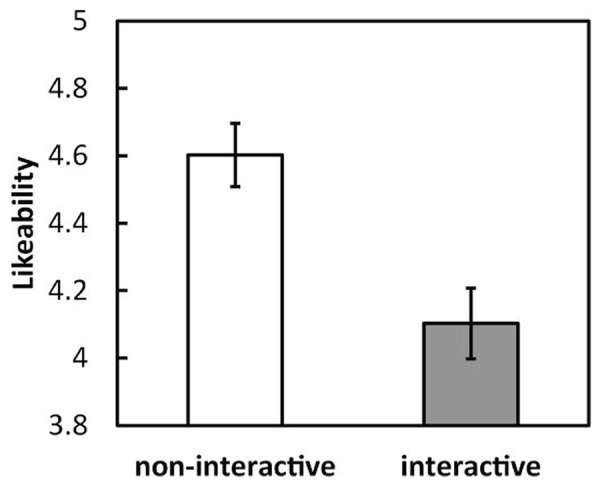
**Mean likeability ratings for all images under each condition (interactive and non-interactive) in Experiment 1.** Error bars indicate SE.

The mean likeability ratings given for all images were calculated for each participant under each condition and then averaged across participants (**Figure 1**). A between-subjects *t*-test revealed that mean likeability under the interactive condition was significantly lower than that under the non-interactive condition [*t*(58) = 3.48, *p* < 0.001, *r* = 0.42]^1^.

#### Differences between devalued and non-devalued images

**FIGURE 2:**
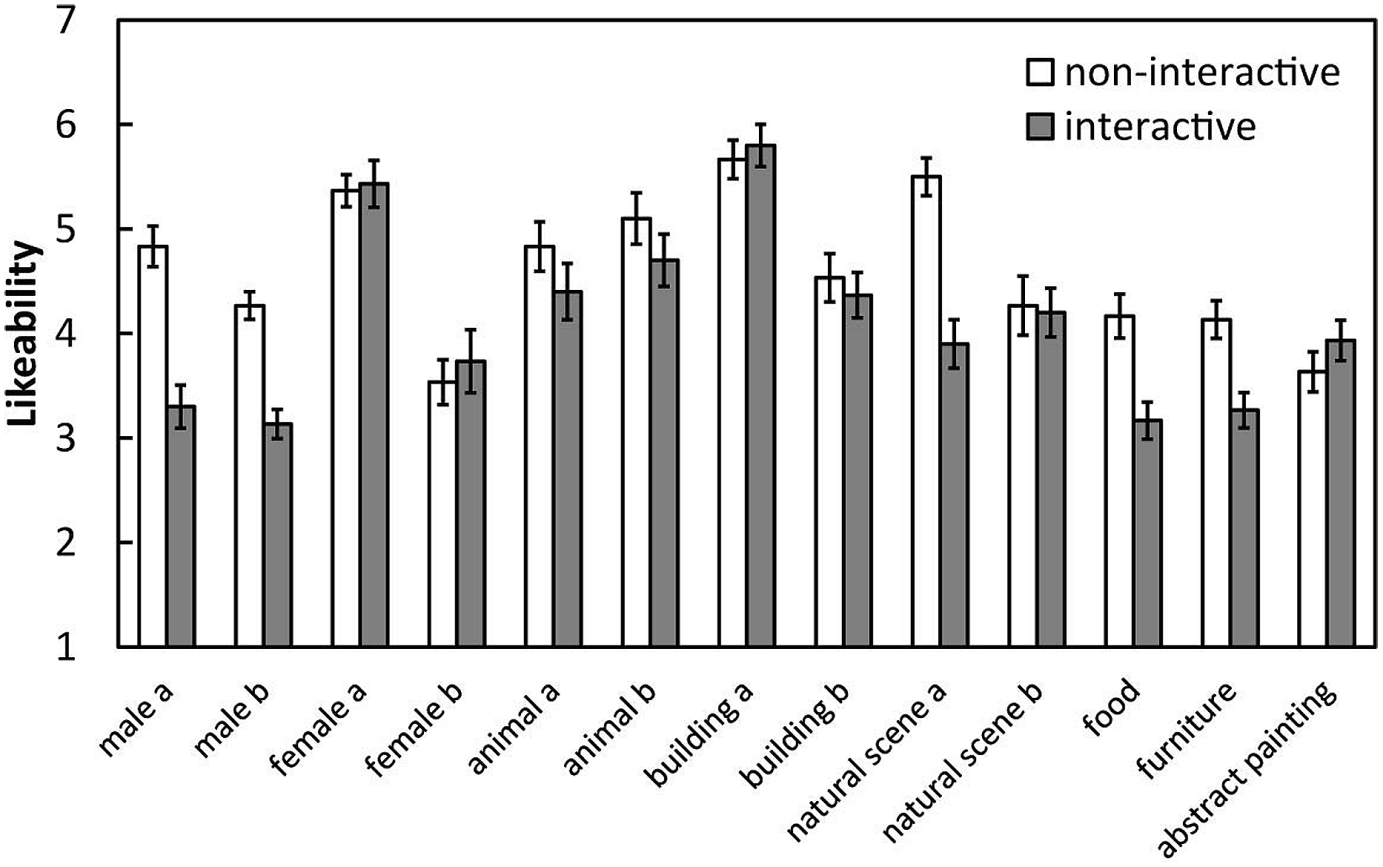
**Mean likeability ratings for each image under each condition in Experiment 1.** Error bars indicate SE.

Mean likeability was calculated separately for each image (**Figure 2**). A 2 (condition: interactive and non-interactive) × 13 (image: 13 images) two-way analysis of variance (ANOVA) revealed significant main effects of condition [*F*(1,58) = 12.14, *p* < 0.001, η^2^ = 0.55] and image [*F*(12,696) = 23.98, *p* < 0.001, η^2^ = 0.32] as well as a significant interaction between these factors [*F*(12,696) = 5.44, *p* < 0.001, η^2^ = 0.07], demonstrating that the decrease in the likeability ratings in the interactive condition relative to the non-interactive condition was modulated by image. The simple main effect of image was significant for five images: male faces a and b, natural scene a, food, and furniture. That is, the likeability ratings for these five images were significantly lower under the interactive compared with the non-interactive condition (*devalued images*). However, the likeability of the other eight images was not significantly different between conditions (*non-devalued images*).

**FIGURE 3:**
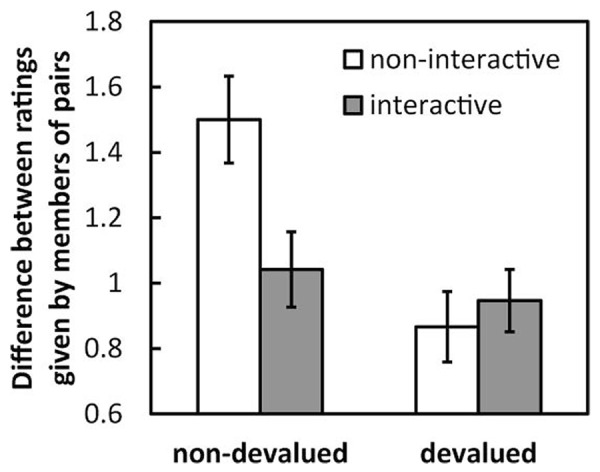
**Mean difference (absolute values) in the ratings given by pairs under each condition and for each image type in Experiment 1.** Error bars indicate SE.

To clarify the differences between devalued and non-devalued images, the mean differences (absolute value) of the ratings given for devalued and non-devalued images by both members of the pairs were calculated and then averaged across pairs (**Figure 3**). A 2 (condition: interactive and non-interactive) × 2 (image type: non-devalued and devalued) two-way ANOVA revealed a significant main effect of image type [*F*(1,28) = 12.82, *p* < 0.005, η^2^ = 0.13] but no effect of condition [*F*(1,28) = 2.07, *n.s.*, η^2^ = 0.04]. There was a significant interaction between these factors [*F*(1,28) = 7.00, *p* < 0.05, η^2^ = 0.07]. Under the non-interactive condition, the difference was larger for non-devalued images than for devalued images [*F*(1,28) = 19.38, *p* < 0.001], and of the non-devalued images, the difference under the non-interactive condition was greater than that under the interactive condition [*F*(1,56) = 7.60, *p* < 0.01].

Furthermore, the conversation data recorded during the experiment was analyzed. Two raters who were naïve about the purpose of this study individually heard the audio data and rated how often negative statements were delivered by either member of the pair; for each pair, this was done for the first half (15 s) and last half (15 s) of the 30-s session, using a score of 0–10 (i.e., 0 indicating that negative statements were not delivered at all; 10 indicating that all statements were negative). As the ratings between the two raters showed a high positive correlation (*r* = 0.83, *p* < 0.01), I considered the ratings as reliable, and took their averages as negativity scores. The mean negativity scores for devalued and non-devalued images were calculated and then averaged across pairs (**Table 1**). A within-subject 2 (image type: non-devalued and devalued) × 2 (period: first half and last half) two-way ANOVA revealed no significant main effects of image type [*F*(1,14) = 3.89, *n.s.*, η^2^ = 0.13] or period [*F*(1,14) = 0.19, *n.s.*, η^2^ = 0.00]. There was a significant interaction between these factors [*F*(1,14) = 6.00, *p* < 0.05, η^2^ = 0.08]. For devalued images, negative information was delivered significantly more often by either member of the pairs in the last half than in the first half [*F*(1,28) = 4.20, *p* < 0.01], though there was no significant difference for non-devalued images [*F*(1,28) = 2.09, *n.s.*].

**Table 1:** Mean negativity scores for non-devalued and devalued images in the first and last half of the sessions in Experiment 1.

	First half	Last half
Non-devalued	5.06	4.77
Devalued	5.17	5.58

### DISCUSSION

Although participants evaluated identical images under both conditions, likeability was generally lower under the interactive condition than under the non-interactive condition. The interactive evaluation influenced the likeability ratings of objects even though preferences were individually recorded without discussion or agreement between partners.

Furthermore, the decrease in likeability under the interactive condition was especially pronounced for images that elicited relatively consistent evaluations across individuals. This is consistent with my prediction derived from the previous studies (Brodbeck et al., 2002; Schulz-Hardt et al., 2006), which indicated that the effect of shared information was stronger when group members held the same initial preferences than when they held different preferences. In fact, the analysis of the contents of the discussion revealed that for devalued images, negative information was increasingly delivered (or shared) by either member of the pairs as the discussion progressed, though this was not the case for non-devalued images in Experiment 1.

Although the mean likeability of the non-devalued images did not differ between the interactive and non-interactive conditions, the differences between the ratings given by the individual members of a pair were smaller under the interactive condition than under the non-interactive condition. This suggests that the exchange of impressions affected individual preferences not only for the devalued but also for the non-devalued images; the effect was different between devalued and non-devalued images. Thus, when group members interactively discuss objects and then independently convey their preferences, co-evaluation affects their decisions anyway.

It has been suggested that group discussions usually begin with members’ exchanging their initial preferences (e.g., Gigone and Hastie, 1993, 1997). Differences in initial opinions should make members of tight-knit groups aware that conflict exists (Festinger, 1964). This, in turn, would be expected to elicit the threat in social interactions, which group members would be motivated to reduce. In fact, when two individuals have different impressions (or attitudes toward the likeability) of an object, they are motivated to compromise with each other to establish or protect a positive relationship (Hahn and Hwang, 1999). Additionally, many previous social psychological studies have shown that individuals tend to match their beliefs and attitudes to those of other people (e.g., Turner, 1991). In accordance with this mechanism, participants in Experiment 1 would be strongly motivated to maintain the daily relationship with their friends after realizing that their attitudes toward the non-devalued image diverged.

Interestingly, differences in the likeability ratings for devalued images given by the members of a pair were equally low under both the interactive and non-interactive conditions; however, the interactive evaluators rated the images as less likeable than did the non-interactive evaluators, irrespective of image category and the degree of likeability of the images. It is suggested here that this new phenomenon may be referred to as the *social-devaluation effect.* The present study is the first to demonstrate the specific direction in which the attitudes of individuals shift following the exchange of impressions. In other words, the expressed likeability of objects generally decreased (never increased) when two participants engaged in social interaction regarding their reactions to an object.

It is possible that negative information is more likely to be shared and/or to elicit greater attention when initial preferences are shared during co-evaluation, and it is also likely that this may have a negative influence on individuals’ likeability ratings of devalued images. When two individuals have similar impressions (or attitudes toward the likeability) of an object, their daily relationship (or social connectedness) is already ensured because similar attitudes lead to the formation of positive bonds between individuals (e.g., Singh and Ho, 2000; Morry, 2007). It has been suggested that individuals want to establish and maintain a stable framework for an ongoing relationship once social connectedness has been ensured (Baumeister and Leary, 1995). That is, they are motivated to maintain and reinforce their ensured relationship by further modulating their attitudes to be in line with ingroup attitudes (Aronson et al., 2010). Once an ingroup is ensured of a positive relationship among members of the group, individuals are eager to boost their own self-esteem as members of that group. Given that this process occurs in social contexts, negative evaluators are seen as more intelligent and competent than are positive evaluators (Amabile, 1983; Gibson and Oberlander, 2008), and a negativity bias would be expected to occur in favor of one’s own group. In fact, a negative evaluative bias in the service of boosting self-esteem is robustly seen in consumer evaluations of products (e.g., Herr et al., 1991; Schlosser, 2005); for example, consumers are influenced only by others’ negative information and, thus, adjust their attitudes downward. In this context of negative bias, evaluators would synergistically increase their self-esteem by interacting with one another to reinforce their daily relationship (cf. Rubin and Hewstone, 1998).

Experiment 1 demonstrated that, under the interactive condition, independently generated likeability ratings of objects are influenced by the exchange of impressions and are dependent on whether initial preferences were similar (devalued images; the social-devaluation effect) or dissimilar (non-devalued images; the convergence of likeability). As discussed above, it is presumable that the influence of shared information is based on the daily relationships among group members, as all pairs in Experiment 1 consisted of friends. If a daily relationship of this sort is necessary for co-evaluation to influence individual preferences, then the effects observed in Experiment 1 would be eliminated in pairs consisting of strangers. This was examined in Experiment 2.

## EXPERIMENT 2

### METHOD

The sample consisted of 30 pairs of naïve volunteers (60 undergraduates in total, 11 males, 18–27 years old); all pairs consisted of participants who were strangers to each other. This was the only difference from Experiment 1.

### RESULTS

**FIGURE 4:**
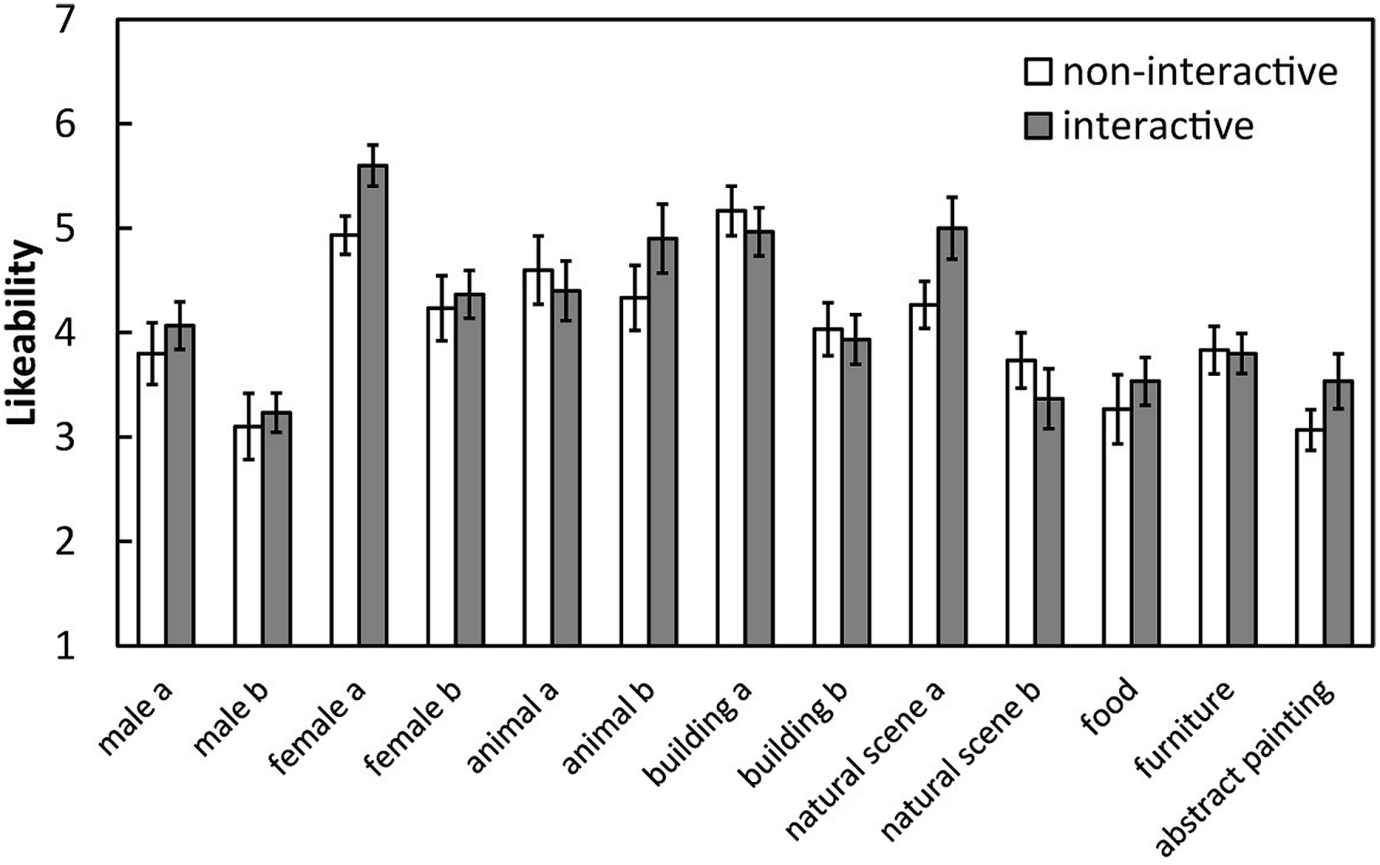
**Mean likeability ratings for each image under each condition in Experiment 2.** Error bars indicate SE.

Mean likeability was calculated separately for each image (**Figure 4**). A 2 (condition: interactive and non-interactive) × 13 (image: 13 images) two-way ANOVA revealed a significant main effect of image [*F*(12,696) = 14.55, *p* < 0.001. η^2^ = 0.17] but not of condition [*F*(1,58) = 1.57, *n.s.*, η^2^ = 0.00]. The interaction between these factors was not significant [*F*(12,696) = 1.00, *n.s.*, η^2^ = 0.01].

**FIGURE 5:**
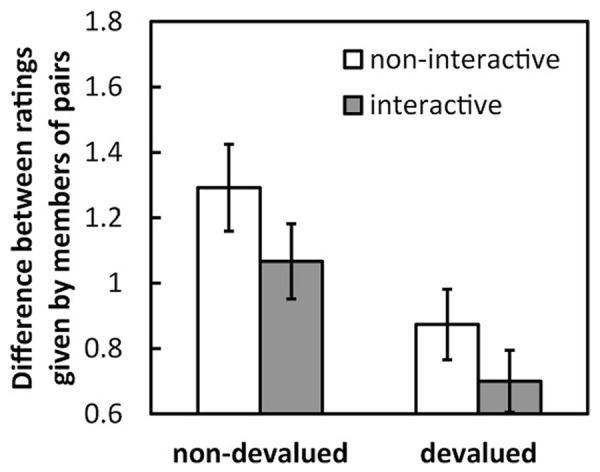
**Mean difference (absolute values) in the ratings given by pairs under each condition and for each image type in Experiment 2.** Error bars indicate SE.

The mean differences (in absolute values) in the ratings given to devalued and non-devalued images, labeled in Experiment 1, by the two members of the pairs were averaged across pairs (**Figure 5**). A 2 (condition: interactive and non-interactive) × 2 (image type: devalued and non-devalued) two-way ANOVA revealed a significant main effect of image type [*F*(1,28) = 11.12, *p* < 0.005, η^2^ = 0.13] but not of condition [*F*(1,28) = 1.86, *n.s.*, η^2^ = 0.03]. The interaction between the factors was not significant [*F*(1,28) = 0.05, *n.s.*, η^2^ = 0.00].

Although they were strangers, all pairs discussed the images until the end of the 30-s session. The conversation data recorded during the experiment was analyzed in a manner similar to Experiment 1. Again, as the ratings between the two raters showed a high positive correlation (*r* = 0.81, *p* < 0.01), I considered the ratings as reliable, and took their averages as negativity scores. The mean negativity scores for devalued and non-devalued images were calculated and then averaged across pairs (**Table 2**). A within-subject 2 (image type: non-devalued and devalued) × 2 (period: first half and last half) two-way ANOVA revealed no significant main effects of image type [*F*(1,14) = 2.02, *n.s.*, η^2^ = 0.04] or period [*F*(1,14) = 0.10, *n.s.*, η^2^ = 0.00]. There was no significant interaction between these factors [*F*(1,14) = 0.00, *n.s.*, η^2^ = 0.00].

**Table 2:** Mean negativity scores for non-devalued and devalued images in the first and last half of the sessions in Experiment 2.

	First half	Last half
Non-devalued	4.70	4.80
Devalued	5.01	5.10

### DISCUSSION

Consistent with the results of Experiment 1, the non-devalued images identified in Experiment 1 again elicited relatively inconsistent evaluations across participants, whereas the devalued images elicited consistent evaluations. However, neither the convergence of likeability via interactive discussion nor the social-devaluation effect was observed in Experiment 2. Also, the frequency of negative information delivered did not change from the first to last periods of the session for either the devalued or non-devalued images in Experiment 2. Therefore, it can be concluded that the influence exerted on the individual likeability of objects by the exchange of impressions is strongly at the foundation of the daily, or already established, relationships between group members.

## GENERAL DISCUSSION

This study aimed to reveal the nature of the co-evaluation of likeability by demonstrating its effect on individual judgments regarding various target images. In Experiment 1, where all pairs of participants consisted of friends, the likeability ratings given to the objects when the participants exchanged their impressions of the objects (interactive condition) were compared with those when the participants did not exchange their impressions (non-interactive condition). Two types of influence of co-evaluation on individual preferences were observed. First, for images that usually elicit relatively inconsistent impressions across individuals (i.e., non-devalued images), the likeability ratings showed greater convergence under the interactive condition, even though the mean likeability ratings given by the pair did not change. Second, for the images that usually elicit relatively consistent impressions across individuals (i.e., devalued images), the likeability ratings decreased under the interactive condition, illustrating the social-devaluation effect. However, in Experiment 2, these effects were eliminated when the participant pairs consisted of strangers. Therefore, the effects of social interactions on individual likeability for objects are strongly predicated on the existence of a daily relationship.

The current findings cannot be explained by previous accounts of the cognitive consequences of affect, such as the feelings-as-information theory (Schwarz and Clore, 1983; Schwarz, 2012), which posits that people rely on their current feelings as diagnostic information when making an affective evaluation of a target. If such a process had occurred in this study, co-evaluation with a stranger, which might be a bit uncomfortable discussion, would have led to a negative emotional state for the participants in Experiment 2, thereby leading to a devaluation in likeability. However, this was not the case; the devaluation occurred via co-evaluation with a friend in Experiment 1.

### THE EFFECT OF EXCHANGING IMPRESSIONS

Presumably, when two individuals have different impressions (or attitudes toward the likeability) of an object, they would be motivated to compromise with each other to establish a positive relationship (or to reduce the threat in social interactions). In this study, this process was observed in response to non-devalued images. On the other hand, when two individuals have similar impressions (or attitudes toward the likeability) of an object, social connectedness should have been ensured. Because individuals want to maintain a stable framework for an ongoing relationship once social connectedness has been ensured (Baumeister and Leary, 1995), they might be motivated to reinforce the ensured relationship by further modulating their attitudes to be in line with ingroup attitudes (Aronson et al., 2010). Consequently, evaluators would synergistically increase their self-esteem by interacting with one another to reinforce their relationship (cf. Rubin and Hewstone, 1998). In fact, a negative evaluative bias in the service of boosting self-esteem is often seen in consumer evaluations of products (e.g., Herr et al., 1991; Schlosser, 2005). Therefore, in this study, the likeability of the devalued images, which tend to elicit consistent evaluations across individuals, was modulated in a negative direction by co-evaluation.

A possible explanation for the social-devaluation effect awaits further investigation. However, these findings newly and obviously demonstrated that the exchange of impressions during co-evaluation modulates the expressed likeability of objects even though participants were told in advance that they did not have to agree with each other and even though their responses were shielded from each other. Interestingly, the effect of co-evaluation differed based on the image type, i.e., whether consistent or inconsistent evaluations were elicited across individuals. It is also noteworthy that already established relationships underlie the effects of co-evaluation on the individual expression of preferences.

### BEYOND CONFORMITY?

It has been shown that individuals tend to match their beliefs and attitudes with those of other people (e.g., Turner, 1991). For example, a recent study reported that the likeability ratings given to objects were modulated by the evaluations of others (Zaki et al., 2011). In that experiment, participants rated the likeability of human faces in the context of information about the likeability ratings given by peers. Participants changed their likeability ratings (and the neural activities in relevant brain areas) to conform to those of their peers. Following this conformity effect, individuals tend to change their ratings of likeability of objects as a function of the attitudes of others toward those objects (see also, Klucharev et al., 2009). In the absence of social interaction, evaluators have to find a way to make their ratings consistent with those of others to achieve an experience of social connectedness with those who are not present; this conformity effect is analogous to the compromises observed in this study in response to the non-devalued images. However, in a co-evaluation situation such as in this study, once social connectedness is ensured, participants can proceed to the next step and develop an interpersonal relationship that serves to establish a stable framework for an ongoing connection (Baumeister and Leary, 1995). Thus, the social-devaluation effect may illuminate cognitive processes that go beyond those that underpin conformity by demonstrating the devaluation of likeability ratings triggered by social interactions.

### FUTURE WORK

Working from a classical sociological view, Sumner (1906) posited that humans, by their very nature, are a species that join together in groups. Also, the concept of a “social brain” holds that the human brain has evolved primarily to enable human beings to have rich, complex social lives on a continuous basis (Dunbar, 1993, 1996). Based on these, I predict that the social-devaluation effect is a culture-free phenomenon. At present, I can conclude that the effect occurs for Japanese participants who are thought to belong to a collectivistic culture (e.g., Rosenstone, 1988; Markus and Kitayama, 1991; Eisenstadt, 1996; Hofstede and Hofstede, 2004). In the future, it is necessary to investigate the effect with participants from an individualistic culture, such as the USA or Europe. At the same time, there is a possibility that the degree of the effect might be different between Eastern and Western cultures. Because Americans are likely to endorse the expression of emotion more than Japanese (Matsumoto et al., 2005), the social-devaluation effect will be strongly observed with Americans, who are expected to deliver more negative statements during the discussion. Furthermore, considering Freud’s (1930) assertion of the need for interpersonal contact in childhood and the attachment theory that assumes that a human needs to form and maintain relationships (Bowlby, 1969, 1973), it would be interesting to investigate the effect with a developmental approach.

## CONCLUSION

The present study newly revealed fundamental and critical effects of co-evaluation with others, such as occurs in realistic social situations, on the likeability ratings given to objects by individuals. When two individuals harbor different impressions of an object, they are motivated to reduce the difference so that they can establish (or recover) an interpersonal relationship. On the other hand, when two individuals have similar impressions of an object, they may be motivated to devalue the object to boost their self-esteem so that they can maintain the stability of their relationship (the social-devaluation effect). That is, interactive evaluation may dynamically and immediately form and maintain social connectedness. Interestingly, the co-evaluation of objects serves to reinforce one’s daily relationships with friends, even though they express their preferences independently and without agreement. In sum, likeability is not merely a matter of individual taste but may be a flexible and adaptive mechanism that fosters human sociality.

## Conflict of Interest Statement

The author declares that the research was conducted in the absence of any commercial or financial relationships that could be construed as a potential conflict of interest.

## Footnotes

1 To investigate the influence of the non-interactive participant, I have run an additional experiment in which the non-interactive condition was conducted entirely with individual participants (the individual condition, *N* = 30). A between-subjects one-way ANOVA that combined the data in the new experiment with the data from Experiment 1 (conditions: individual, interactive, and non-interactive) revealed a significant main effect of condition [*F*(2,87) = 6.24, *p* < 0.005, η^2^ = 0.13]. The likeability ratings in the individual condition (*M* = 4.51, SD = 0.62) were significantly different from those in the interactive condition (*M* = 4.10, SD = 0.58) but were not significantly different from those in the non-interactive condition (*M* = 4.60, SD = 0.52). These results demonstrate that the effect in Experiment 1 can be attributed to verbal social interaction, not the mere presence of others.
